# The risk of offspring mood and anxiety disorders in the context of prenatal maternal somatic diseases: a systematic review and meta-analysis

**DOI:** 10.1017/S2045796021000706

**Published:** 2022-01-12

**Authors:** Xiayu Gong, Zhixin Fan, Hanfang Xu, Hanzhang Wang, Ningxi Zeng, Ling Li, Lili Wu, Can Yan

**Affiliations:** Research Center for Basic Integrative Medicine, Guangzhou University of Chinese Medicine, Guangzhou 510006, China

**Keywords:** Anxiety disorders, intrauterine environment, mood disorders, somatic diseases

## Abstract

**Aims:**

The importance of prenatal maternal somatic diseases for offspring mood and anxiety disorders may be overlooked or undervalued. We conducted the first systematic review and meta-analysis assessing the risk of offspring mood and anxiety disorders in the context of prenatal maternal somatic diseases.

**Methods:**

We screened articles indexed in Embase (including Embase, MEDLINE, PubMed-not-MEDLINE), PsycARTICLES and PsycINFO databases up to August 2021. 21 studies were included. We examined the overall associations between prenatal maternal somatic diseases and offspring mood/anxiety disorders. Analyses were stratified according to maternal somatic diseases and follow-up duration.

**Results:**

We observed an increased risk of mood and anxiety disorders in the context of prenatal maternal somatic diseases [relative risk (RR) = 1.26; 95% confidence interval (CI) 1.15–1.37, RR = 1.31; 95% CI 1.24–1.38]; maternal obesity(RR = 1.92; 95% CI 1.72–2.11), hypertensive disorders (RR = 1.49; 95% CI 1.11–1.86) and infertility (RR = 1.26, 95% CI 1.14–1.39) were risk factors for mood disorders; maternal polycystic ovary syndrome (RR = 1.61; 95% CI 1.42–1.80), severe obesity (RR = 1.56; 95% CI 1.44–1.68) and moderate obesity (RR = 1.36; 95% CI 1.28–1.44) were risk factors for anxiety disorders. Prenatal maternal somatic diseases increased the risk of mood disorders in childhood and adulthood (RR = 1.71; 95% CI 1.34–2.09/RR = 1.19; 95% CI 1.09–1.30), as well as the risk of anxiety disorders in adulthood (RR = 1.33; 95% CI 1.26–1.41).

**Conclusion:**

The results indicate that prenatal maternal somatic diseases are associated with offspring mood and anxiety disorders, and that the associations may be long-lasting.

## Introduction

Mood disorders (bipolar and depressive disorders) and anxiety disorders (separation anxiety disorder, selective mutism, specific phobias, social anxiety disorder, panic disorder, agoraphobia and generalised anxiety disorder) are complex mental disorders resulting from multiple factors such as genetic predisposition, parenting style, family environment, socioeconomic status. Fetal origins of mental disorders have been attracting increasing attention. Awareness of prenatal risk factors is crucial for prevention strategies.

Prenatal maternal health plays an important role in the subsequent mental health of offspring (O'Donnell and Meaney, 2017). A relationship has been established between prenatal maternal psychosocial factors (psychological stress, anxiety and depression) and psychiatric disorders in offspring (Robinson *et al*., 2019; Su *et al*., 2021). In addition to psychosocial factors, somatic diseases are also common among pregnant women. A cohort study including more than 1.3 million childbirths showed that the prevalence of maternal chronic diseases during pregnancy was 15.76%, with somatic diseases accounting for about 70% (Jolving *et al*., 2016). In the context of somatic diseases, hormones, metabolites, cytokines and nutrients are altered, which may implicate an adverse intrauterine environment and affect fetal neurodevelopment (Mac Giollabhui *et al*., 2019; Nattero-Chavez *et al*., 2019; Lu-Culligan and Iwasaki, 2020). Furthermore, some maternal somatic diseases alter the normal composition of breast milk (Tekin Guler *et al*., 2021) and severe somatic diseases could influence family environment. These could contribute to the risk of offspring mood/anxiety disorders.

Accumulating high-quality cohort studies have assessed the impact of prenatal maternal somatic diseases on mood and anxiety disorders in offspring, but the results are not consistent. To our knowledge, there have been no systematic review and meta-analysis assessing the risk of offspring mood and anxiety disorders in the context of prenatal maternal somatic diseases. The importance of prenatal maternal somatic diseases for offspring mood and anxiety disorders may be overlooked or undervalued. The relationships between them merit further investigation. The results could provide valuable information for exploring underlying mechanisms of mood and anxiety disorders, individualised risk prediction models, and targeted interventions during or before pregnancy. To fill this gap, this systematic review and meta-analysis aimed to assess the risk of offspring mood and anxiety disorders in the context of prenatal maternal somatic diseases.

## Methods

This study was conducted in accordance with Preferred Reporting Items for Systematic Reviews (PRISMA) (Moher *et al*., 2009). PRISMA checklist was presented in online Supplementary materials. The protocol was registered in PROSPERO (CRD42021241441).

### Search strategy

We searched the following databases for relevant literature written in English from their inception until 31 August 2021: Embase (including Embase, MEDLINE, PubMed-not-MEDLINE), PsycARTICLES (via EBSCO) and PsycINFO (via EBSCO). The search strategy consisted of relevant Medical Subject Heading (MeSH) terms, Emtree term-exploded, keywords and word variants. The detailed search strategies for each database were fully described in online Supplementary Table S1. In addition, we searched the reference lists of all included studies, related reviews for further potential studies.

### Inclusion and exclusion criteria

Articles were eligible if they met the following criteria: (1) exposures: offspring were exposed to maternal somatic diseases *in utero* (somatic diseases diagnosed during pregnancy or chronic somatic diseases diagnosed before pregnancy; somatic diseases included all non-psychiatric diseases), (2) control group(s): had a comparison group(s) without the exposure(s), (3) outcomes: included mood or anxiety disorders diagnosed according to any recognised diagnostic criteria or self-report (mood disorders included bipolar and depressive disorders; anxiety disorders included separation anxiety disorder, selective mutism, specific phobias, social anxiety disorder, panic disorder, agoraphobia and generalised anxiety disorder), (4) statistical indicators were provided to examine the effect of prenatal maternal somatic diseases on mood or anxiety disorders in offspring, (5) study design: cohort studies, including population-based cohort studies and registry-based studies.

Articles were excluded if they met the following criteria: (1) exposed group was mixed with maternal somatic diseases diagnosed postpartum; (2) reviews, meta-analyses, abstracts or conference proceedings.

When there were multiple groups of useful data in one article, only the data derived from the group with the largest sample size or the most severe exposure (e.g. in moderate and severe obesity, the data of severe obesity were pooled) were selected for the meta-analysis. In addition, we did not delete articles that presented overlapping samples as they included different maternal somatic diseases or outcomes, but only the data from the largest sample size was used in the meta-analysis.

### Selection of the studies

After removing duplications, titles and abstracts were reviewed independently by two researchers for initial screening, and then full text. Any disagreement was resolved through group discussions. Endnote was used as the bibliographic software.

### Data extraction and quality assessment

Two researchers independently extracted data. Any disagreement was resolved through group discussions. The following data were extracted: author, year of publication, the country where the study was conducted, sample and source, exposure and measure, outcome and measure, adjusted confounders, and measure of association. All included articles were assessed in terms of methodological quality according to the Newcastle–Ottawa Quality Assessment Scale (NOS) for cohort studies. NOS consists of eight items across three domains: selection (four items, each with one star), comparability (one item, with a maximum of two stars) and outcome (three items, each with one star). Studies were graded as good quality (7–9 stars), fair (4–6 stars) and poor (0–4 stars).

### Statistical analysis

The method was based on the relative risk (RR) with 95% confidence intervals (CIs) obtained in each study. If the RR was not reported, the hazard ratio (HR)/odds ratio (OR) is considered to be approximately equal to RR. RRs fully adjusted were preferentially pooled in our analyses. Cochrane *Q* test and *I*^2^ statistics were used to evaluate the heterogeneity. A fixed-effects model was adopted when *I*^2^ < 50%, otherwise, a random-effects model was used. Funnel plot, trim and fill method and Egger test were used to detect potential publication bias. To assess the stability of the meta-analysis, sensitivity analysis was performed. Sensitivity analysis was performed by excluding studies one by one to explore the impact of each study on the overall results. Furthermore, analyses were stratified according to maternal somatic diseases and follow-up duration (or offspring's age at diagnosis). Quantitative meta-analysis was conducted for an outcome when more than one study presented relevant data. The reason why we adopted stratified analysis rather than traditional subgroup analysis was that the former can make better use of the data in our study. Analyses were performed with Stata 16.0.

## Results

**Fig. 1.**
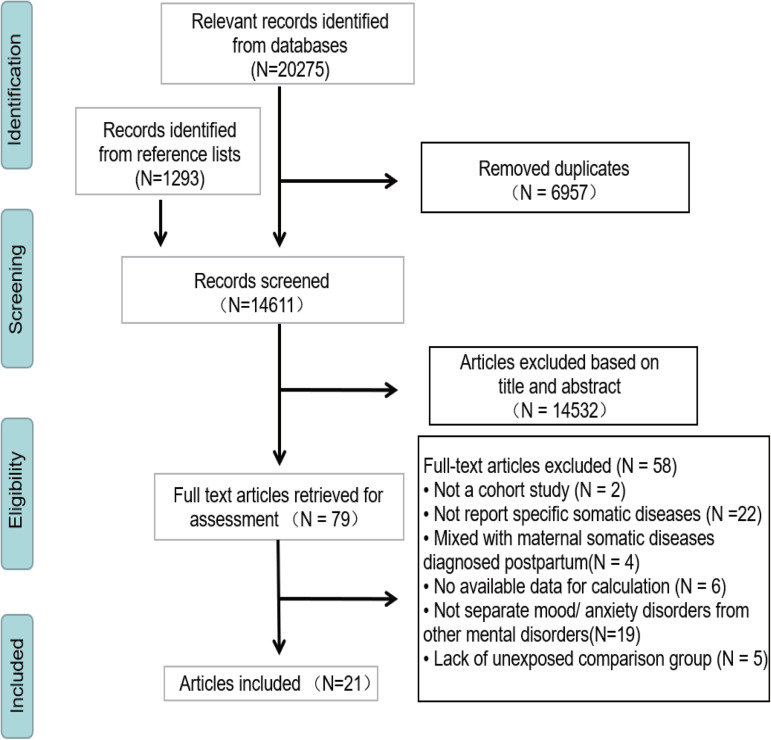
Study selection process.

In total, 21 articles were eligible for inclusion (Pang *et al*., 2009; Tuovinen *et al*., 2012, 2014; Robinson *et al*., 2013, 2020*a*, 2020*b*; Betts *et al*., 2015; Kingston *et al*., 2015; Svahn *et al*., 2015; Jolving *et al*., 2018*a*, 2018*b*; Rochat *et al*., 2018; Dachew *et al*., 2019, 2020; Lydholm *et al*., 2019; Momen *et al*., 2019; Wu *et al*., 2019; Chen *et al*., 2020; Kong *et al*., 2020; Wang *et al*., 2020, 2021). A summary of the study selection process was presented in Fig. 1. Table 1 shows the general characteristics of the included studies. More than 5.7 million offspring were involved, and the sample size ranged from 778 to 2 412 721 participants. Twelve maternal somatic diseases were involved, infections (bacterial and viral infections), hypertensive disorders (chronic hypertension, gestational hypertension and preeclampsia), diabetes (type 2 diabetes, insulin-treated pregestational diabetes and gestational diabetes), thyroid diseases (Graves' disease and Hashimoto's thyroiditis), obesity [body mass index (BMI) ⩾ 30], polycystic ovary syndrome (PCOS and hirsutism), infertility, cancer, asthma, rheumatoid arthritis, hyperemesis gravidarum, migraine. According to the Newcastle–Ottawa Scale, 15 studies were graded as good quality and six were graded as fair quality (online Supplementary Table S2).

**Table 1.** Characteristics of included studies

Author, publication year	Country	Sample (source)	Exposure (measure)	Outcome (measure)	Adjusted confounders
Betts *et al*. (2015)	Australia	*N* = 2439 (All pregnant public patients attending consecutive obstetric clinic visits at the Mater Misericordiae Hospital in Brisbane between 1981 and 1984)	Maternal infection (self-report)	Major depressive disorders, generalised anxiety disorders, social and specific phobias, and panic disorders (DSM-IV)	Child's gender, birth weight, maternal age, antenatal anxiety, antenatal depression, previous births, maternal smoking, maternal alcohol use, maternal education
Chen *et al*. (2020)	Finland	*N* = 1 105 997 (All live births during 1996–2014 in Finland, registered in the Drugs and Pregnancy Database)	Maternal polycystic ovary syndrome, anovulatory infertility and obesity (ICD-9, ICD-10)	Mood and anxiety disorders (ICD-10)	Maternal age at delivery, parity, country of birth, mother married at birth, smoking during pregnancy, diagnoses of systemic inflammatory disorders, psychiatric disorders, purchase of N05 and N06 during pregnancy
Dachew *et al*. (2020)	UK	*N* = 6739 (Data came from the Avon Longitudinal Study of Parents and Children)	Hypertensive disorders of pregnancy (International Society for the Study of Hypertension in Pregnancy)	Depression (DSM-IV, ICD-10)	Maternal age, parity, maternal alcohol use during the first 3 months of pregnancy, maternal smoking during the first 3 months of pregnancy, and maternal depression and anxiety during pregnancy
Dachew *et al*. (2019)	UK	*N* = 5231 (Data came from the Avon Longitudinal Study of Parents and Children)	Hypertensive disorders of pregnancy, pregnancy diabetes (International Society for the Study of Hypertension in Pregnancy)	Anxiety disorders (DSM-IV, ICD-10)	Maternal education status, social class, marital status, ethnicity, maternal age at delivery, parity, maternal alcohol use and smoking during pregnancy, maternal pregnancy diabetes status, maternal pre-pregnancy BMI, maternal depression and anxiety during pregnancy, child's sex, gestational age at delivery, and birthweight
Jolving *et al*. (2018*a*)	Denmark	*N* = 1 560 955 (All live born children in Denmark from 1989 to 2013)	Maternal thyroid disease (ICD-8, ICD-10)	Mood disorders (ICD-8, ICD-10)	Sex of the child, year of birth, maternal age at birth, delivery mode, multiple birth, birth order, preterm birth, small for gestational age, maternal mood disorders
Jolving et al. (2018*b*)	Denmark	*N* = 1 380 645 (All live born children in Denmark from 1989 to 2013)	Maternal rheumatoid arthritis (ICD-8, ICD-10)	Mood disorders (ICD-8, ICD-10)	Not performed adjusted analysis on the result of mood disorders
Kingston *et al*. (2015)	Canada	*N* = 19 316 (Data came from the Population Health Research Data Repository at the Manitoba Centre for Health Policy, University of Manitoba)	Maternal hypertension, diabetes (ICD-9, ICD-10)	Anxiety (ICD-9, ICD-10)	Maternal age, completed high school, on income assistance, neighbourhood income quintile, relationship status, married, partnered, parity, caesarean delivery, antepartum haemorrhage, social isolation, relationship distress, prenatal psychological distress, postnatal psychological distress, psychological distress in early childhood, prenatal substance use, infant sex, 5 min Apgar score, preterm, small for gestational age, breastfeeding initiation
Kong *et al*. (2020)	Finland	*N* = 647 099 (All live births between 2004 and 2014 in Finland)	Maternal obesity, insulin-treated pregestational diabetes, type 2 diabetes, gestational diabetes (ICD-10)	Mood disorders, anxiety disorders (ICD-10)	Offspring birth year, sex, perinatal problems, number of foetuses, caesarean delivery, maternal age group at delivery, parity, mother's marital status at birth, mother's country of birth, maternal smoking, maternal psychiatric disorder, maternal use of psychotropic medication during pregnancy and maternal systemic inflammatory disease
Lydholm *et al*. (2019)	Denmark	*N* = 1 206 600 (All children born in Denmark between 1 July 1996 and 31 December 2015)	Maternal infections (medical records)	Mood disorders (ICD-10)	Sex, birth year, concurrent infection in the other parent, parental infections outside the time period, parental level of education at childbirth, parental age at childbirth, parental physical illnesses at childbirth, any parental psychiatric diagnoses at childbirth
Momen *et al*. (2019)	Denmark	*N* = 2 158 430 (All children born from 1 January 1978 to 31 December 2012 in Denmark)	Maternal cancer (ICD-7, ICD-10)	Mood disorders (ICD-8, ICD-10)	Sex, maternal age at time of birth, child's birth year, maternal highest education at time of childbirth, parity and parental diagnosis of a mental or behavioural disorder
Pang *et al*. (2009)	UK	*N* = 6152 (Data came from a cohort born in the UK between 1946 and 1980, another cohort was from the UK National Health Service Central Register)	Maternal prenatal viral infection (medical records)	Depression (ICD-9)	Birthdate, sex and area of birth
Robinson *et al*. (2013)	Australia	*N* = 2868 (Data came from the Western Australian Pregnancy Cohort Study)	Maternal pre-pregnancy obesity (self-report)	Mood disorders (DSM-IV, The 118-item Child Behavior Checklist for ages 4–18)	Maternal age, maternal education, presence of the biological father in the family home, family income, stress in pregnancy, maternal cigarette smoking, maternal alcohol consumption, gestational age at birth, birth weight, hypertensive diseases of pregnancy, gestational diabetes and length of breastfeeding
Robinson *et al*. (2020*a*)	USA	*N* = 1915 (Data came from the Upstate KIDS study)	Maternal polycystic ovary syndrome, hirsutism (vital records, self-report)	Anxiety disorders (maternal reported diagnosis)	Maternal age, race, education, insurance status, marital status, smoking, father's age difference, maternal and paternal history of affective disorder, maternal body mass index, child's sex
Robinson *et al*. (2020*b*)	USA	*N* = 1915 (Data came from the Upstate KIDS study)	Maternal pre-pregnancy obesity (vital records, self-report)	Anxiety disorders (maternal reported diagnosis)	Maternal age, race, education, insurance status, marital status, smoking, father's age difference, maternal and paternal history of affective disorder, maternal body mass index, child's sex
Rochat *et al*. (2018)	Africa	*N* = 1256 (Data came from the Siyakhula Cohort, which includes HIV-negative children, enrolled from the Africa Health Research Institute)	Maternal HIV-positive during pregnancy (medical records)	Mood disorders, anxiety disorders (DSM, Child Behavior Checklist)	Mother/caregiver age and education, mother's employment and relationship status, indicators of mother/caregiver clinical depression and anxiety; number of resident adults and children in household and exposure to crime; child HIV exposure, age and sex, early feeding, if the child ever repeated a school grade, food insecurity, and receipt of a child grant; whether the primary caregiver was the mother and clinical parenting stress subscale indicators
Svahn *et al*. (2015)	Denmark	*N* = 2 412 721 (All children born in Denmark between 1969 and 2006)	Maternal infertility (ICD-8, ICD-10)	Mood disorders (ICD-8, ICD-10)	Year of birth, birth order (1, 2, ⩾3), sex, maternal age at birth (⩽25, 26–28, 29–32, ⩾33 years), paternal age at birth (⩽26, 27–30, 31–34, ⩾35 years) and parental history of mental disorder
Tuovinen *et al*. (2012)	Finland	*N* = 5970 (Data came from the Helsinki Birth Cohort Study)	Gestational and chronic hypertension, preeclampsia (medical records)	Mood disorders (ICD-8, ICD-9, ICD-10)	Gestational age, weight at birth, father's occupational status in childhood, parity, mother's age and BMI at delivery
Tuovinen *et al*. (2014)	Finland	*N* = 778 (Data came from the Helsinki Birth Cohort Study)	Gestational and chronic hypertension, preeclampsia (the criteria of the National High Blood Pressure Education Program Working Group on High Blood Pressure in Pregnancy)	Depression, anxiety (DSM)	Sex, year of birth, gestational age, weight for gestational age, head circumference at birth, placental weight, father's occupational status in subject's childhood, parity, mother's age, BMI at delivery, breastfeeding, own maximum level of education in adulthood and age at completion of the questionnaire
Wang *et al*. (2020)	Denmark	*N* = 2 092 897 (All singleton live births born in Denmark during 1978–2012)	Hyperemesis gravidarum (ICD-8, ICD-10)	Separation anxiety disorder (emotional disorders) (ICD-8, ICD-10)	Children's age at time scale, sex, year of birth, parity, parental age at birth, maternal education level, maternal country of origin, maternal cohabitation and parental psychiatry disorders
Wang *et al*. (2021)	Denmark	*N* = 2 069 785 (All singleton live births born in Denmark during 1978–2012)	Maternal migraine (ICD-8, ICD-10)	Mood disorders (ICD-8, ICD-10)	Sex, birth yea, parity, maternal characteristic (age, education level, origin, cohabitation, cardiovascular diseases), paternal age, parental psychiatric disorders before the childbirth
Wu *et al*. (2019)	Sweden	*N* = 2 258 098 (All children born in Sweden from 1973 to 1995)	Maternal asthma (Medical Birth Register)	Bipolar disorder [(Jolving et al., 2018*a*, 2018*b*; Wang *et al*., 2021) ICD-8, ICD-9, ICD-10]	Age, sex, calendar year, socioeconomic status, urban born, mother Swedish born, birth order, mother's hospitalisation due to infection during pregnancy, parental serious mental illness, maternal age, paternal age and other parent's asthma status before birth

ICD, International Classification of Diseases of the World Health Organization; DSM, Diagnostic and Statistical Manual of Mental Disorders; BMI, body mass index; N05 (antipsychotics, anxiolytics, hypnotics and sedatives), N06 (antidepressants, psychostimulants and nootropics).

### The risk of offspring mood disorders in the context of prenatal maternal somatic diseases

**Fig. 2.**
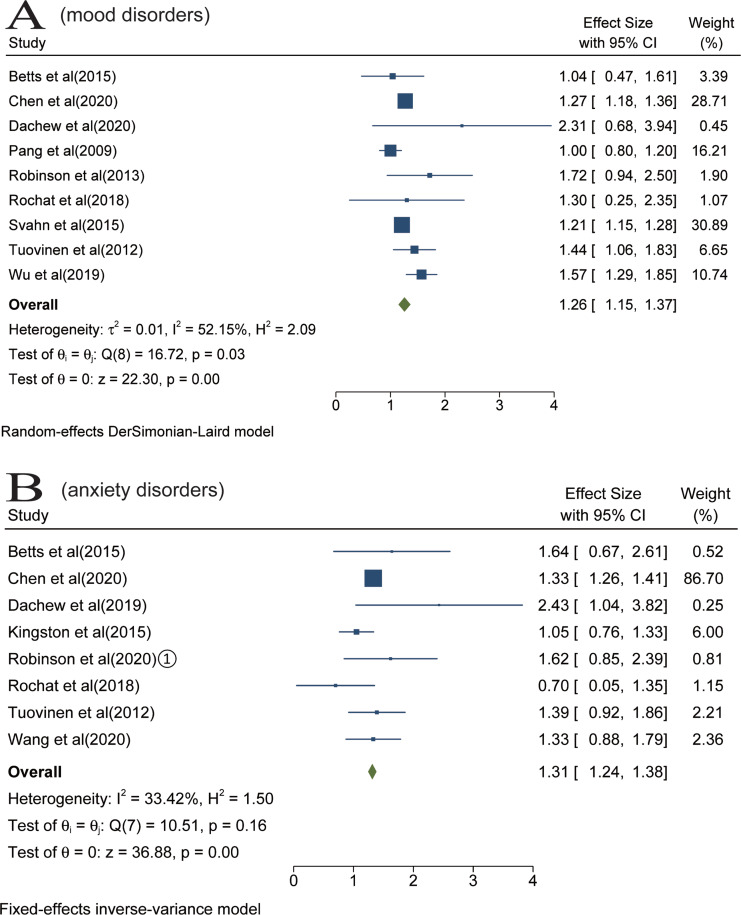
Results of overall meta-analysis [Forest plot presenting combined effect estimates; ES, effect size (relative risk)]. (a) The risk of offspring mood disorders in the context of prenatal maternal somatic diseases. (B) The risk of offspring anxiety disorders in the context of prenatal maternal somatic diseases.

We identified 16 studies that examined the association of maternal somatic diseases with mood disorders (Pang *et al*., 2009; Tuovinen *et al*., 2012, 2014; Robinson *et al*., 2013; Betts *et al*., 2015; Svahn *et al*., 2015; Jolving *et al*., 2018*a*, 2018*b*; Rochat *et al*., 2018; Lydholm *et al*., 2019; Momen *et al*., 2019; Wu *et al*., 2019; Chen *et al*., 2020; Dachew *et al*., 2020; Kong *et al*., 2020; Wang *et al*., 2021). Data of seven studies were not be pooled because they used overlapping populations (Tuovinen *et al*., 2014; Jolving *et al*., 2018*a*, 2018*b*; Lydholm *et al*., 2019; Momen *et al*., 2019; Kong *et al*., 2020; Wang *et al*., 2021). The meta-analysis of nine studies suggested that the risk of offspring mood disorders increased in the context of prenatal maternal somatic diseases, with a pooled RR of 1.26 (95% CI 1.15–1.37) based on a random-effects model. High heterogeneity was observed (*I*^2^ = 52%, *p* < 0.05) (Fig. 2a).

**Fig. 3.**
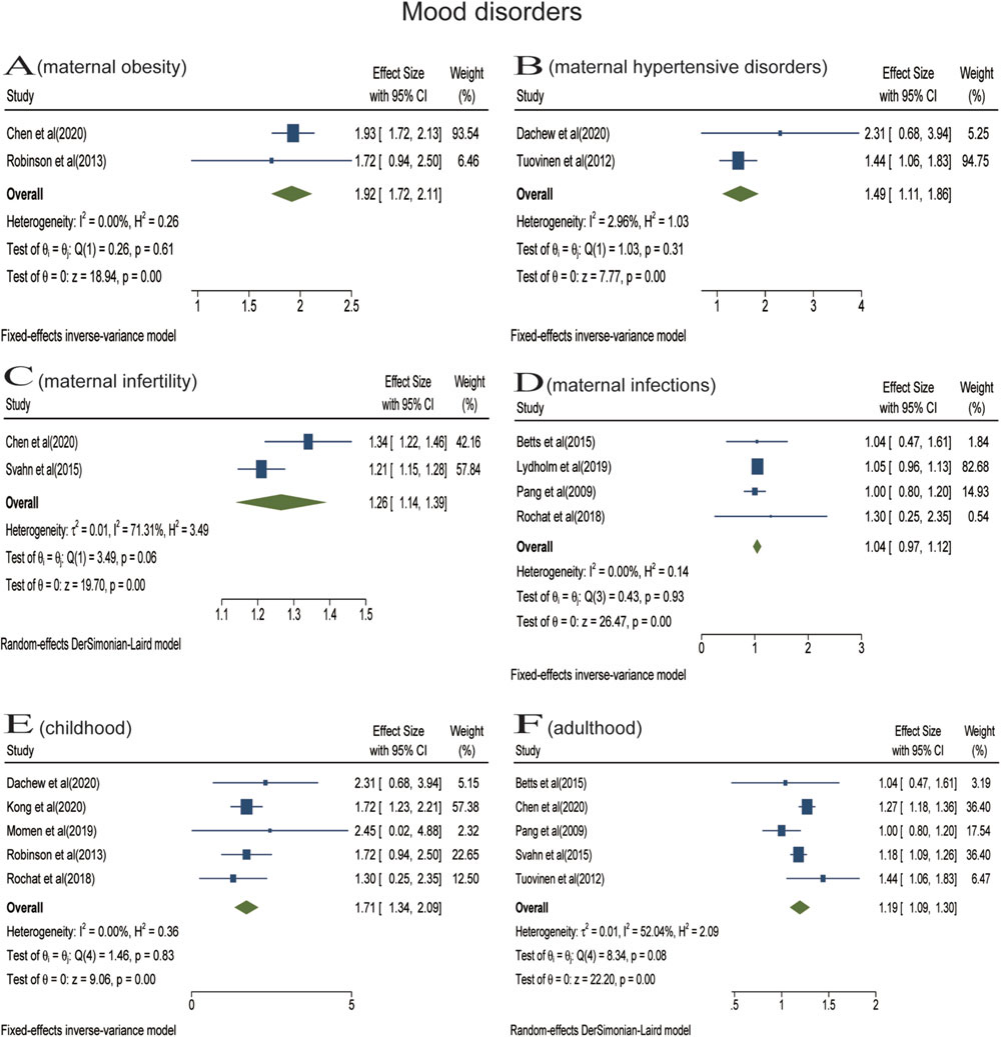
Stratified analyses of mood disorders [Forest plot presenting combined effect estimates; ES, effect size (relative risk)]. (a) The association between maternal obesity and mood disorders in offspring. (b) The association between maternal hypertensive disorders and mood disorders in offspring. (c) The association between maternal infertility and mood disorders in offspring. (d) The association between maternal infections and mood disorders in offspring. (e) The association between maternal somatic diseases and mood disorders in childhood. (f) The association between maternal somatic diseases and mood disorders in adulthood.

Analyses were stratified according to maternal somatic diseases. Pooled effect of two studies showed that maternal obesity was significantly associated with offspring mood disorders (RR = 1.92; 95% CI 1.72–2.11) (Fig. 3a). In the meta-analysis of two studies, maternal hypertensive disorders increased the risk of offspring mood disorders (RR = 1.49; 95% CI 1.11–1.86) (Fig. 3b). Pooled effect of two studies suggested that the risk of offspring mood disorders increased in the context of maternal infertility (RR = 1.26; 95% CI 1.14–1.39) (Fig. 3c). The meta-analysis of four studies found that there was no statistical association between prenatal maternal infections and offspring mood disorders (RR = 1.04; 95% CI 0.97–1.12) (Fig. 3d).

In the stratified analyses, we did not perform meta-analyses for maternal PCOS, cancer, asthma, diabetes, thyroid diseases and rheumatoid arthritis due to the limited number of eligible studies. These studies reported that prenatal exposure to maternal PCOS (Chen *et al*., 2020), diabetes with BMI ⩾ 35, cancer (Momen *et al*., 2019), asthma (Wu *et al*., 2019) and migraine (Wang *et al*., 2021) elevated the risk of mood disorders, while maternal diabetes with BMI < 30 (Kong *et al*., 2020), thyroid diseases (Jolving *et al*., 2018*b*) and rheumatoid arthritis (Jolving *et al*., 2018*a*) were not associated with mood disorders.

Analyses were also stratified according to follow-up duration (or offspring's age at diagnosis), including childhood (aged < 18 years) and adulthood (aged ⩾ 18 years). In the meta-analysis of five studies, prenatal maternal somatic diseases were associated with the increased risk for mood disorders in childhood (RR = 1.71; 95% CI 1.34–2.09) (Fig. 3e). The meta-analysis including five studies found that prenatal maternal somatic diseases increased the risk of mood disorders in adulthood (RR = 1.19; 95% CI 1.09–1.30) (Fig. 3f). Only one article reported the impact on older offspring (aged 69.3 ± 3.1 years) and found that maternal hypertensive disorders during pregnancy (HDP) increased the risk of offspring depressive disorders seven decades later (Tuovinen *et al*., 2014).

### The risk of offspring anxiety disorders in the context of prenatal maternal somatic diseases

Ten studies examined the association between maternal somatic diseases and anxiety disorders (Tuovinen *et al*., 2014; Betts *et al*., 2015; Kingston *et al*., 2015; Rochat *et al*., 2018; Dachew *et al*., 2019; Chen *et al*., 2020; Kong *et al*., 2020; Robinson *et al*., 2020*a*, 2020*b*; Wang *et al*., 2020). Data of two studies were not be pooled because they used overlapping populations (Kong *et al*., 2020; Robinson *et al*., 2020*b*). The meta-analysis of eight studies suggested that the risk of anxiety disorders increased in the context of prenatal maternal somatic diseases, with a pooled RR of 1.31 (95% CI 1.24–1.38) based on a fixed-effects model. There was no evidence of significant heterogeneity among the studies (*I*^2^ = 33%, *p* > 0.05) (Fig. 2b).

Analyses were stratified according to maternal somatic diseases. Pooled effect of two studies showed that the risk of offspring anxiety disorders significantly increased in the context of maternal PCOS (RR = 1.61; 95% CI 1.42–1.80) (Fig. 3a). In the meta-analysis of two studies, maternal severe obesity (BMI ⩾ 35) increased the risk of offspring anxiety disorders (RR = 1.56; 95% CI 1.44–1.68) (Fig. 3b). Pooled effect of two studies suggested that maternal moderate obesity (30 ⩽ BMI < 35) was associated with offspring anxiety disorders (RR = 1.36; 95% CI 1.28–1.44) (Fig. 3c). The meta-analysis of three studies found that there was no statistical association between prenatal maternal hypertensive disorders and offspring anxiety disorders (RR = 1.32; 95% CI 0.84−2.11) (Fig. 3d). The meta-analysis including two studies found that prenatal maternal infections did not increase the risk of offspring's anxiety disorders (RR = 1.10; 95% CI 0.19–2.01) (Fig. 3e). The meta-analysis did not find a statistical association between prenatal maternal diabetes and anxiety disorders of offspring among three studies (RR = 1.00; 95% CI 0.88–1.13) (Fig. 3f).

In the stratified analyses, we did not perform a meta-analysis for maternal infertility and hyperemesis gravidarum due to the limited number of eligible studies. One study reported a significant association between maternal infertility and anxiety disorders of offspring (Chen *et al*., 2020), the other found no significant association between maternal hyperemesis gravidarum and separation anxiety disorder (emotional disorders) in offspring (Wang *et al*., 2020).

Analyses were also stratified according to follow-up duration (or offspring's age at diagnosis). In the meta-analysis of five studies, prenatal maternal somatic diseases were not associated with offspring anxiety disorders in childhood (RR = 1.28; 95% CI 0.99–1.57) (Fig. 3g). The meta-analysis including three studies found that prenatal maternal somatic diseases increased the risk of anxiety disorders in adulthood (RR = 1.33; 95% CI 1.26–1.41) (Fig. 3h). Only one article reported the impact on older offspring and found that maternal HDP increased the risk of anxiety (Tuovinen *et al*., 2014).

### Publication bias and sensitivity analysis

**Fig. 4.**
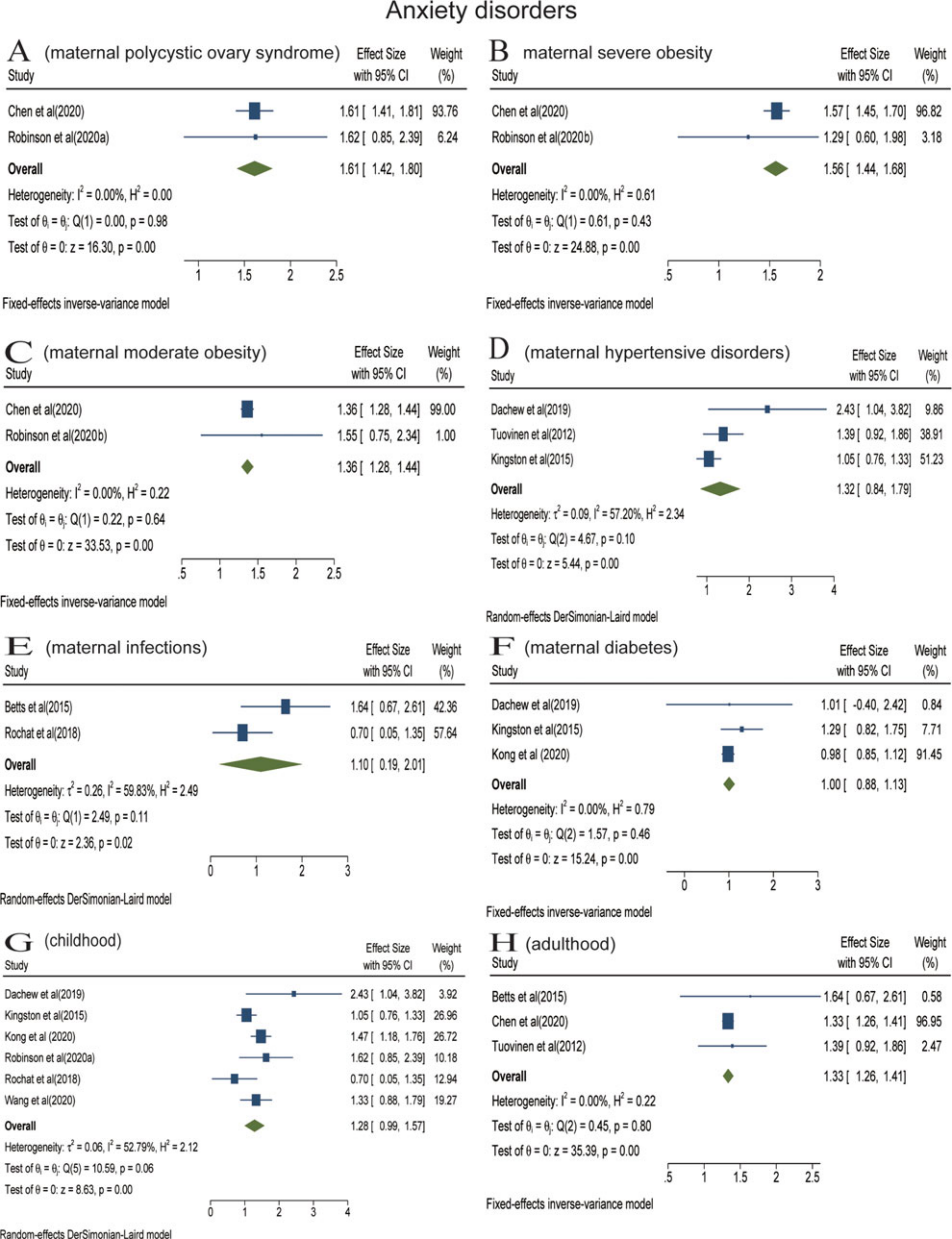
Stratified analyses of anxiety disorders [Forest plot presenting combined effect estimates; ES, effect size (relative risk)]. (a) The association between maternal polycystic ovary syndrome and anxiety disorders in offspring. (b) The association between maternal severe obesity and anxiety disorders in offspring. (c) The association between maternal moderate obesity and anxiety disorders in offspring. (d) The association between maternal hypertensive disorders and anxiety disorders in offspring. (e) The association between maternal infections and anxiety disorders in offspring. (f) The association between maternal diabetes and anxiety disorders in offspring. (g) The association between maternal somatic diseases and anxiety disorders in childhood. (h) The association between maternal somatic diseases and mood disorders in adulthood.

Due to the small number of studies after stratified analyses, funnel plot and Egger test were only calculated for the main results of mood and anxiety disorders. Although no evidence of publication bias was found based on Egger test (*p* > 0.05), the asymmetric funnel plot suggested the possibility of publication bias (online Supplementary Fig. S1). The ‘trim and fill’ method was then used to recalculate the pooled results. The adjusted pooled effect of mood disorders exhibited a similar trend with two potential studies filled (RR = 1.24; 95% CI 1.13–1.36) (online Supplementary Fig. S1). The result for anxiety disorders was not altered with two potential studies filled (RR = 1.24; 95% CI 1.13–1.36) (Fig. 4d).

Sensitivity analysis did not significantly alter these findings, indicating that our results were relatively stable (online Supplementary Fig. S2).

## Discussion

To our knowledge, this is the first systematic review and meta-analysis assessing the risk of offspring mood and anxiety disorders in the context of prenatal maternal somatic diseases. The overall meta-analyses confirmed that prenatal maternal somatic diseases were associated with an increased risk of mood and anxiety disorders in offspring. For mood disorders, we identified that maternal obesity, hypertensive disorders, and infertility were risk factors. For anxiety disorders, risk factors were maternal PCOS and obesity. Furthermore, our study emphasised the impact of prenatal maternal somatic diseases on mood and anxiety disorders may be long-lasting.

The overarching finding of the meta-analysis showed that prenatal maternal somatic diseases were related to offspring mood/anxiety disorders. Mood and anxiety disorders are complex mental disorders, the RRs generally <2 indicated that they each modulated risk by a relatively small. Prenatal exposure to both maternal somatic diseases and psychiatric factors implicates an adverse intrauterine environment, but they might point toward differing underlying mechanisms of an increased risk of mood/anxiety disorders. Although most of the included studies (16 of 21) adjusted maternal psychiatric disorders or distress, the effect of maternal psychosocial factors cannot be excluded, as a relationship has been reported early between somatic diseases and mental health (Verhaak, 1997; Harter *et al*., 2007). The results of our meta-analysis provided evidence for prenatal origins of mood and anxiety disorders. Nonetheless, some prenatal maternal somatic diseases have an indirect effect on their offspring after childbirth. This association could be mediated through children's perceived stress [e.g. cancer (Osborn, 2007)], household income, and socioeconomic status [e.g. diabetes (Kim *et al*., 2015)] that are relevant to maternal somatic diseases.

Prenatal maternal obesity was associated with offspring mood and anxiety disorders in the results. Obesity diagnosed before pregnancy was also included because obesity is considered to be a chronic inflammatory disease (De Lorenzo *et al*., 2019). Previous studies have found that maternal obesity is associated with an increased risk of neuropsychiatric disorders, basically consistent with our findings (Rivera *et al*., 2015). The findings are also supported by animal studies, which observed that maternal obesity (high-fat diet model) increased offspring anxiety and depression-like behaviours (Sullivan *et al*., 2010; Gawlinska *et al*., 2021). Maternal obesity is characterised by elevated systemic levels of nutrients (fatty acids, glucose), hormones (leptin, insulin) and inflammatory markers (C-reactive protein, interleukin and tumour necrosis factor) (Gil-Campos *et al*., 2004; Challier *et al*., 2008; Farah *et al*., 2012). The pathological metabolic states and chronic inflammatory conditions influence fetal neurodevelopment (Sureshchandra *et al*., 2018; Baud and Berkane, 2019), which may mediate the associations.

Notably, although maternal inflammation during pregnancy has been associated with offspring psychiatric disorders (Han *et al*., 2021), we did not find that exposure to maternal infections was associated with the risk of subsequent mood and anxiety disorders. The previous review also failed to find a definitive link between maternal infections and mood disorders in offspring (Simanek and Meier, 2015). However, a recent meta-analysis found that maternal infection exposure was related to the risk of offspring psychosis (Zhou *et al*., 2021), and a nested case-control study reported a nearly fourfold increase in the risk of bipolar disorder after exposure to maternal influenza at any time during pregnancy (Parboosing *et al*., 2013). The inconsistent results may suggest that the magnitude, duration and composition of inflammatory signals determine how the offspring are ultimately affected (St-Germain *et al*., 2020). Future studies are needed to investigate the differences of various inflammation-related diseases during pregnancy and how they affect fetal brain programming.

The results showed that hypertensive disorders during pregnancy (HDP) were associated with an increased risk of mood disorders in offspring, but the association with anxiety disorders was not statistically significant. Although a previous meta-analysis found that preeclampsia was associated with an elevated risk of offspring schizophrenia, the association with other psychiatric disorders was inconclusive (Dachew *et al*., 2018). A case-control study involving 333 participants reported inconsistent results, with no significant differences in the prevalence of maternal preeclampsia between adult offspring with mood disorders and healthy controls (Pugliese *et al*., 2019). This inconsistency might be attributable to different exposure definitions, study methodology and sample sizes. The underlying mechanism of this association may be related to placental ischemia, hypoxia, inflammation and fetal programming of the hypothalamic-pituitary-adrenal axis in the context of HDP (Vitoratos *et al*., 2012; Henley *et al*., 2016; Sharma *et al*., 2018; Socha *et al*., 2020). In addition, HDP predicts an increased risk of preterm delivery, small for gestational age and low birth weight (Avorgbedor *et al*., 2019; Thakur and Dangal, 2020; Poudel *et al*., 2021), which may partly mediate the association between HDP and offspring mood disorders (Su *et al*., 2021).

Offspring exposure to maternal PCOS was associated with an increased risk of anxiety disorders in the meta-analysis, consistent with the results of animal studies (Hu *et al*., 2015; Risal *et al*., 2021). Elevated circulating androgen is a characteristic clinical feature of PCOS, which hypothetically could be related to an increased risk of offspring anxiety disorders (Lombardo *et al*., 2012; Risal *et al*., 2021). In addition, women with PCOS are more likely to seek help from assisted reproductive treatment due to common subfertility. Moreover, PCOS is associated with high rates of pregnancy complications and perinatal problems (Teede *et al*., 2018). Assisted reproductive treatment, pregnancy complications and perinatal problems are risk factors for psychosis (Davies *et al*., 2020; Rissanen *et al*., 2020), which may partially mediate the association.

Children born to women with infertility were at an increased risk of developing mood disorders in our study. Although the birth of offspring seems to mean that infertility has been effectively treated, preexisting pathological conditions of infertility may still affect the foetus. For example, PCOS and reproductive inflammation are common causes of female infertility, though successful pregnancy, excess androgens and inflammatory factors could persist during pregnancy and affect fetal brain development. In addition, women with infertility are more likely to have obstetric complications, premature birth and low-birth-weight infants (Thomson *et al*., 2005; Luke *et al*., 2016). These predict an increased risk of mood disorders in offspring. However, we cannot identify the role of fertility treatment in the association. Medications used for fertility treatment are given before or early in pregnancy, thus the embryos may be affected by them, but there is a lack of relevant researches (Koren *et al*., 2020). It has been reported that children conceived by assisted reproductive treatment (ART) are at a higher risk of being diagnosed with autism (Liu *et al*., 2017), mental retardation (Sandin *et al*., 2013) and any other psychiatric disorders (Rissanen *et al*., 2020) than those naturally conceived. It is noteworthy that these control groups were composed of children naturally conceived, rather than children whose parents experienced infertility but did not use ART. Therefore, we are unable to differentiate the role that the fertility treatment played in the association from that of infertility itself. Future studies should further examine the preexisting pathology of infertility and offspring health outcomes.

The study did not find an association between maternal diabetes and anxiety disorders in offspring, which is consistent with the previous systematic reviews (Stahlberg *et al*., 2020). Noticeably, however, that one of the included studies showed that the association became statistically significant when maternal diabetes combined with severe obesity, and was stronger than that of either alone (Kong *et al*., 2020). It may reflect that the combination of diabetes and severe obesity leads to a worse intrauterine environment.

Due to the limited number of studies, maternal thyroid disease, rheumatoid arthritis, asthma, cancer, etc., were not included in the stratified analyses. The impact of these maternal somatic diseases on offspring mood and anxiety disorders deserves further investigation. For example, the association between maternal cancer prenatally diagnosed and mood disorders (HR = 2.45; 95% CI 1.02–5.89) was significantly stronger than those postnatally diagnosed (HR = 1.43; 95% CI 1.14–1.79) in one of our included studies (Momen *et al*., 2019). However, the findings of Chen *et al*. suggested that parental cancer during pregnancy was not associated with the overall risk of mental illness in offspring (Chen *et al*., 2018).

In the context of maternal somatic diseases, the risk of mood disorders was increased in both childhood and adulthood, as was the risk of anxiety disorders in adulthood. The findings suggested that the effects of prenatal maternal somatic diseases were long-lasting and even persist into old age. However, the results should be interpreted with caution because the two ages were not evaluated in the same study.

There are some limitations as well. First, although a total of 21 studies were included, the majority of the studies were from northern European populations, and there was a lack of data from Asian populations. Second, when it came to specific exposures, the number of studies was small, and for some exposures, there were not enough studies to allow quantitative analysis. Third, a pooled risk consistently adjusted for the same variables could not be reached because different variables are adjusted in each study. Forth, different diagnostic criteria and follow-up periods were adopted for the same outcome, which may be the reason for the significant heterogeneity of some results. Fifth, in the context of maternal somatic disease, the effects of the disease itself and treatment measures are included, yet we cannot distinguish their effects in the associations. Sixth, it may be difficult to diagnose children, though mood and anxiety disorders occur at any time throughout the lifespan. And both mood and anxiety disorders may not be diagnosed for a long time after the onset. Therefore, the real risk may be underestimated.

In conclusion, the results of our study indicate that prenatal maternal somatic diseases can be associated with offspring mood and anxiety disorders, and that the associations may be long-lasting. These findings advance the understanding of the prenatal origins of risk for mood and anxiety disorders. More high-quality prospective studies are needed to resolve the limitations mentioned above.

## Supplementary material

For supplementary material accompanying this paper visit http://dx.doi.org/10.1017/S2045796021000706. click here to view supplementary material

## Data

Data extraction tables will be made available to any interested parties on request to the corresponding author.
