# Supplementary material for: The risk of offspring mood and anxiety disorders in the context of prenatal maternal somatic diseases: a systematic review and meta-analysis
Source: Epidemiol Psychiatr Sci. 2022 Jan 12;31:e6. doi: 10.1017/S2045796021000706 (PMC8786618; doi:10.1017/S2045796021000706)

**Supplemental Table S1-2, Figure S1-2**

The risk of offspring mood and anxiety disorders in the context of prenatal maternal somatic diseases: a systematic review and meta-analysis

1. Table S1. Search strategy (Page 2-3)
2. Table S2. Quality assessment according to the Newcastle-Ottawa Scale (Page 4)
3. Figure S1. Funnel plot, trim and fill test (Page 5)
4. Figure S2. Sensitivity analysis (Page 6)

**Table S1. Search strategy**

| **Search strategy for EMBASE** | |
| --- | --- |
| #1 | ‘mood disorders’/exp OR “depressive disorders”/exp OR “cyclothymic disorder”/exp OR “disruptive mood dysregulation disorder”/exp OR “major depressive disorder”/exp OR “persistent depressive disorder”/exp OR “premenstrual dysphoric disorder”/exp OR “bipolar disorder”/exp OR “anxiety disorders”/exp OR “separation anxiety”/exp OR “selective mutism” OR “specific phobia”/exp OR “generalized anxiety disorder”/exp OR “social anxiety disorder”/exp OR “panic disorder”/exp OR agoraphobia/exp OR “generalized anxiety disorder”/exp |
| #2 | (“mood disorder*” OR “affective disorder*” OR “cyclothymic disorder*” OR “cyclothymic personalit*” OR cyclothymia OR depress* OR melancholia* OR “dysthymic disorder*” OR dysthymia OR bipolar OR manic OR affective OR “selective mutism” OR anxiety OR agoraphobia* OR panic OR phobia* OR phobic OR claustrophobia*): ti, ab, kw |
| #3 | #1 OR #2 |
| #4 | “risk factor”/exp |
| #5 | risk$ OR hazard$ OR “odds ratio” OR HR OR RR OR exposure$ OR exposed OR factor$ OR determinant$ |
| #6 | #4 OR #5 |
| #7 | exp “cohort analysis”/ |
| #8 | (cohort* OR Longitudinal OR “follow up” OR prospective*): ti, ab, kw |
| #9 | #7 OR #8 |
| #10 | ((maternal OR mother$ OR parent$ OR parental OR pregnant OR pregnancy OR pregnance OR antenatal* OR prenatal* OR progestation* OR pregestational OR gestation* OR “in utero” OR intrauterine OR woman OR women) AND (progeny OR progenies OR offspring OR daughter$ OR son$ OR child* OR adolescen* OR toddler OR adult* OR youth* OR infant$ OR infancy OR schoolchild* OR kid$)): ti, ab, kw |
| #11 | [english]/lim AND [1-1-1966]/sd NOT [1-9-2021]/sd |
| #12 | #3 AND #6 AND #9 AND #10 AND #11 |
| **Search strategy for PsycArticles and PsyCINFO (via EBSCO)** | |
| #1 | SU (mood disorders OR depressive disorders OR cyclothymic disorder OR disruptive mood dysregulation disorder OR major depressive disorder OR persistent depressive disorder OR premenstrual dysphoric disorder OR bipolar disorder OR anxiety disorders OR separation anxiety OR selective mutism OR specific phobia OR generalized anxiety disorder OR social anxiety disorder OR panic disorder OR agoraphobia OR generalized anxiety disorder) |
| #2 | AB (“mood disorder#” OR “affective disorder#” OR “cyclothymic disorder#” OR “cyclothymic personalit*” OR cyclothymia OR depress* OR mood OR emotion* OR melancholia# OR “dysthymic disorder#” OR dysthymia OR bipolar OR manic OR affective OR “selective mutism” OR anxiety OR anxious OR agoraphobia# OR panic OR phobia# OR phobic OR claustrophobia#) |
| #3 | #1 OR #2 |
| #4 | SU (risk factors) |
| #5 | AB (risk# OR hazard# OR “Odds ratio” OR HR OR RR OR exposure# OR exposed OR factor# OR determinant#) |
| #6 | #4 OR #5 |
| #7 | SU (cohort studies) |
| #8 | AB (cohort# OR Longitudinal* OR “follow up” OR prospective*) |
| #9 | #7 OR #8 |
| #10 | AB (maternal OR mother# OR parent# OR parental OR pregnant OR pregnancy OR pregnance OR antenatal* OR prenatal* OR progestation* OR pregestational OR gestation* OR “in utero” OR intrauterine OR woman OR women) AND AB (progeny OR progenies OR offspring OR daughter# OR son# OR child* OR adolescen* OR toddler OR adult* OR youth* OR infant# OR infancy OR schoolchild* OR kid#) |
| #11 | Limiters-Published Date: -20210831; Population Group: Human; Document Type: Journal Article; English; Exclude Book Reviews; Exclude Non-Article Content |
| #12 | #3 AND #6 AND #9 AND #10 AND #11 |

**Table S2. Quality assessment** **according to the Newcastle-Ottawa Scale**

| Study | Selection | Comparability | Outcome | Total |
| --- | --- | --- | --- | --- |
| Betts et al 2015 | ** | ** | *** | 7 |
| Chen et al 2020 | **** | * | *** | 8 |
| Dachew et al 2020 | **** | * | * | 6 |
| Dachew et al 2019 | **** | ** | * | 7 |
| Jølving et al 2018a | **** | ** | *** | 9 |
| Jølving et al 2018b | **** | / | *** | 7 |
| Kingston et al 2015 | *** | ** | *** | 8 |
| Kong et al 2020 | **** | ** | *** | 9 |
| Lydholm et al 2019 | **** | ** | *** | 9 |
| Momen et al 2019 | **** | ** | *** | 9 |
| Pang et al 2009 | *** | * | *** | 7 |
| Robinson et al 2013 | *** | * | ** | 6 |
| Robinson et al 2020a | ** | ** | * | 5 |
| Robinson et al 2020b | ** | ** | * | 5 |
| Rochat et al 2018 | **** | ** | ** | 8 |
| Svahn et al 2015 | *** | ** | *** | 8 |
| Tuovinen et al 2012 | *** | * | ** | 6 |
| Tuovinen et al 2014 | *** | * | ** | 6 |
| Wang et al 2020 | **** | ** | *** | 9 |
| Wang et al 2021 | **** | ** | *** | 9 |
| Wu et al 2019 | **** | ** | *** | 9 |

Studies were graded as good quality (7-9 stars), fair (4-6 stars), and poor (0-4 stars)

**Figure S1. Funnel plot, trim and fill test.** (A) funnel plot related to mood disorders. (B) funnel plot related to anxiety disorders. (C) trim and fill test related to mood disorders. (D) trim and fill test related to anxiety disorders. Each dot represents one included study. The vertical line indicates the pooled effect size; funnel indicates 95% confidence intervals.


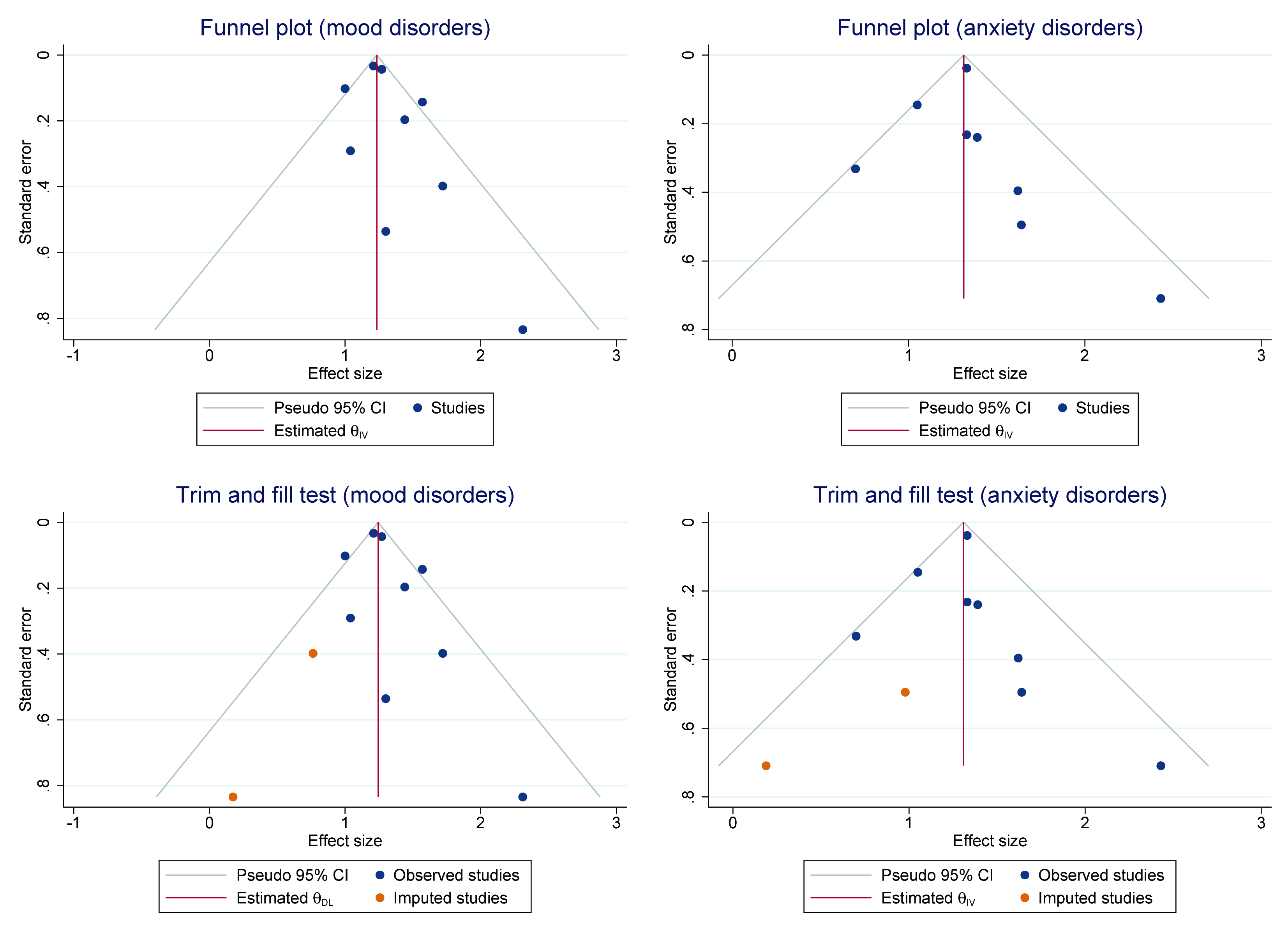


**Figure S2.** **Sensitivity analysis.** (A) sensitivity analysis for mood disorders. (B) sensitivity analysis for anxiety disorders. Estimates when omitting one study at each time.


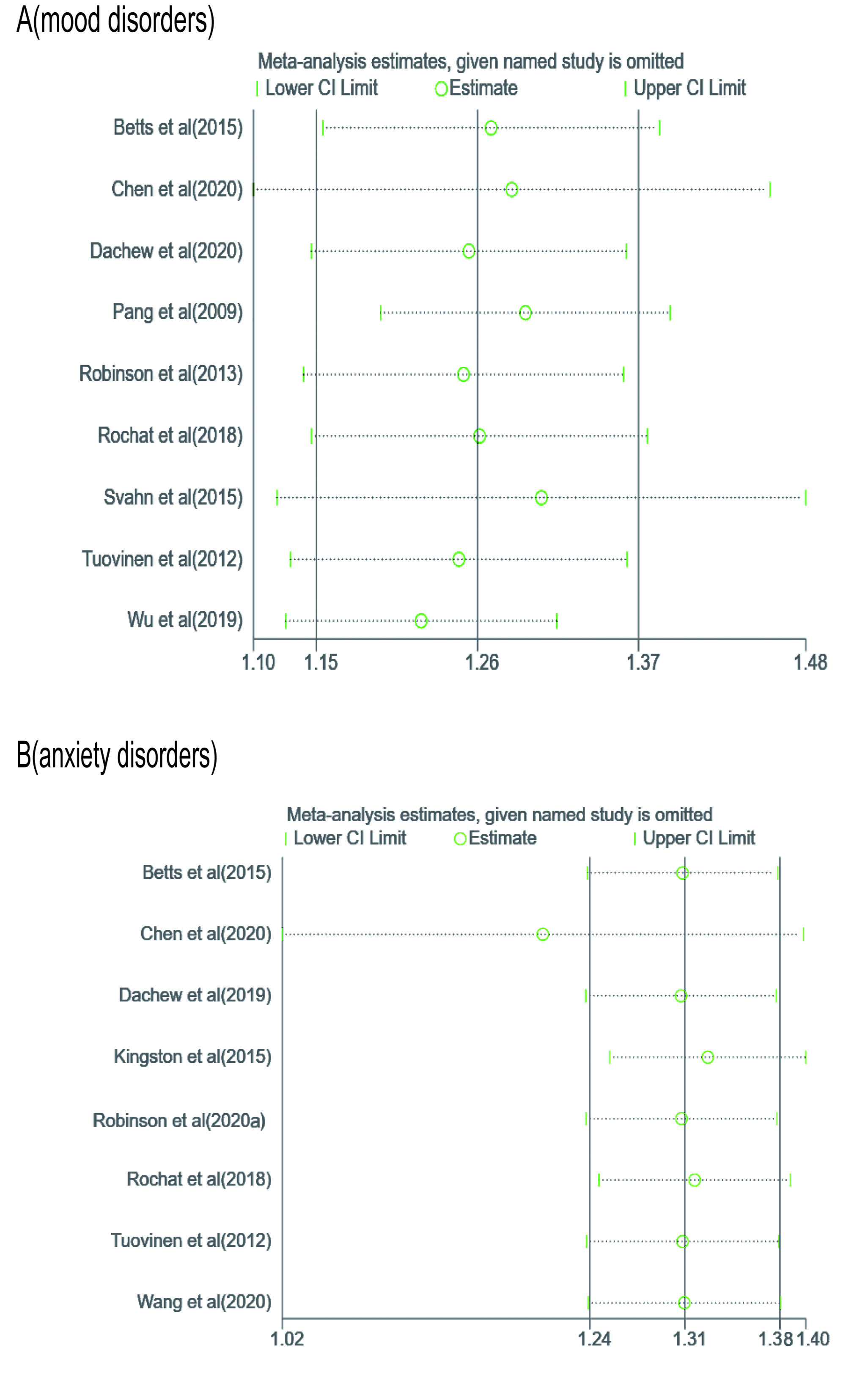

Supplement: Supplementary file 1 [file epssup.zip › S2045796021000706sup003.docx]
